# Targeted Osmotic Lysis: A Novel Approach to Targeted Cancer Therapies

**DOI:** 10.3390/biomedicines10040838

**Published:** 2022-04-02

**Authors:** Harry J. Gould, Dennis Paul

**Affiliations:** 1 Department of Neurology, Louisiana State University Health Sciences Center, New Orleans, LA 70112, USA; 2 Department of Pharmacology and Experimental Therapeutics, Louisiana State University Health Sciences Center, New Orleans, LA 70112, USA

**Keywords:** cancer, targeted therapies, targeted osmotic lysis, pulsed electric fields, advanced stage carcinoma, sodium channels, sodium pumps, Na^+^, K^+^-ATPase

## Abstract

The conventional treatment of cancer has been based on the delivery of non-selective toxins and/or ionizing energy that affect both the cancer and normal tissues in the hope of destroying the offending disease before killing the patient. Unfortunately, resistance often develops to these treatments and patients experience severe, dose-limiting adverse effects that reduce treatment efficacy and compromise quality of life. Recent advances in our knowledge of the biology of tumor cells and their microenvironment, the recognition of surface proteins that are unique to specific cancers and essential to cell growth and survival and signaling pathways associate with invasion and metastasis have led to the development of targeted therapies that are able to identify specific cellular markers and more selectively deliver lethal treatment to the invading cancer thus improving efficacy and limiting adverse effects. In the context of targeted approaches to cancer therapy, we present targeted osmotic lysis as a novel and fundamentally different approach for treating advanced-stage carcinoma that exploits the conserved relationship between voltage-gated sodium channels and Na^+^, K^+^-ATPase and has the potential to increase survival without compromising quality of life in a broad spectrum of highly malignant forms of cancer.

## 1. Perspective on the Disease

Cancer, the mechanisms associated with neoplastic transformation and the growth of malignant tissues have likely co-existed in individual organisms since life began. Malignancies are characterized by an exceptional rate of growth, a high percentage of frequently dividing cells and increased cellular longevity [1]. This enables the cancer cells to compete successfully with the host for essential nutrients and oxygen, eventually resulting in the death of the individual earlier than their genetically programmed longevity. From a Darwinian perspective [2], cancer might be considered a feature of living organisms that evolved to ensure survival of a species in deference to survival of an individual by acting in concert with natural wear and tear as a check on immortality, thus preserving a favorable ecological niche by controlling over population and the over-utilization of available resources. In addition, limiting survival greatly reduces the likelihood that largely unrecognized, debilitating injuries associated with incidental misfortune or self-inflicted environmental injury will weaken the genepool and be passed on to later generations [3,4]. That said, cancer is most often viewed as a scourge to existence, often afflicting individuals during the most productive time in their lives and in so doing, destroys the quality of life for those affected, for their families and for society in general. 

## 2. Early Approaches to Treatment

Because of cancer’s devastating effect on quality of life, scientists and clinicians alike have sought for centuries to identify methods to eliminate or control the negative effects of the disease to improve productivity and preserve quality of life under the mantra, “first, do no harm” [5]. Despite the fact that since the 16th century, we have known that “prevention is better than cure” [6], only the intuitive approaches of self-examination, early detection and avoidance of known carcinogens have been effective methods for prevention.

By contrast, efforts to control the disease have focused on treatments that most effectively eliminate existing disease while sparing as much normal tissue as possible. Instinctively, surgical modalities were the first to be employed to remove identified and debilitating abnormalities with the thought that comorbid impairment of function would return if the underlying reason for the individual’s imminent demise was removed. Although radical surgical intervention increases survival, the procedures are often associated with post-operative morbidity of pain, scarring and disfigurement. Cures are frequently elusive because unidentified micrometastases that remain beyond the borders of a successful resection frequently recur [1,7,8,9]. Nonetheless, surgical intervention remains a significant player in managing cancer of many types.

Following shortly on Wilhelm Roentgen’s discovery of X-ray in 1895 [10], radiotherapy was considered and adopted as a way to treat cancer [9]. Like surgical resection, radiotherapy provided a direct approach to eliminating a cancer and improve survival but could also miss micrometastases and is similarly plagued by undesirable adverse effects related to the collateral damage in surrounding normal tissues that produce significant pain and scarring as well as affecting cardiovascular, pulmonary and gastrointestinal function [11]. Although radiotherapy could, in some instances, affect a cure [12], the realization that exposure to radiation itself could also induce cancer in normal tissues created a need of caution for this mode of therapy for both the patient and the provider of treatment [13].

Direct eradication of cancerous lesions using surgery and radiation improved survival, but disease recurrence was not uncommon. Although disease recurrence was initially disappointing, observations made in the early part of the 20th century, indicated that exposure to certain toxins and alkylating agents could alter cellular function through direct toxic effects or through modification of important cellular maintenance or survival functions could slow growth and reproduction of rapidly growing and dividing cells [9]. Since malignancies were known to be characterized by exceptional growth rate and cell division far exceeding that seen even in normal tissues with a high rate of turnover, the use of nitrogen mustard and a number of other chemical agents were introduced into the treatment regimens for many forms of cancer. Over the years, additional agents were developed and used to reduce tumor burden, and in some cases, eliminate the cancer. Although chemotherapeutic agents showed promise, it was recognized that the non-selective nature of the toxic agents also affected the cells in normal tissues that had high rates of turnover leading to intolerable adverse effects, thereby limiting their use and the coining of the phrase that the hope for chemotherapy was to “be able to kill the cancer before killing the patient” [1,14,15]. In order to mitigate the deleterious effects of these early therapeutic approaches while preserving the benefits, therapies used tissue-sparing approaches to surgical intervention and irradiation and employed adjuvant agents that could enhance the beneficial effects of chemotherapy when provided at lower and less toxic doses [9]. This approach reduced the number of mutilating operations and post-surgical morbidity, post-radiation cosmetic disfigurement and scarring and chemically-induced adverse effects and became the standard of cancer care by the late 1960s [9]. Despite the persistence of unpleasant adverse effects, implementation of these therapeutic options, coupled with education promoting early detection and treatment, led to an increased frequency of cancers that could be cured and a significant improvement in the 5-year survival rate for patients who were diagnosed in the early stages of neoplastic transformation [1,16].

## 3. Immune Therapies

Unfortunately, far less progress had been realized in the treatment of cancers diagnosed late in the disease until the late 1990s with the introduction of the modern age of immune and targeted therapies [1,9]. Following the discovery that tumor growth could be decreased by selectively blocking receptors found to be uniquely expressed in estrogen-receptor positive forms of breast cancer [17], early efforts began to identify receptors unique to cancer cells that play a role in enhancing tumor invasiveness, growth, angioneogenesis for tumor support, and migration for which treatments could be designed and directed to deliver therapeutic agents or toxins for the elimination of essential supportive functions and the selective destruction of a cancer. Unique features of cancer cells were sought that could identify the cancer cells as “non-self” or invaders and a potential threat to survival that could be recognized by the body’s immune system as foreign and thus, targets for removal [18]. Following on the path used to successfully eradicate smallpox and polio [19,20], the technology has made it possible to discover appropriate markers that can be used in the development of vaccines to prevent many forms of cancer. Until prevention becomes a reality, unique cell markers are being isolated and used to produce tumor-specific monoclonal antibodies that locate, recognize and destroy the cells that express the unique markers [9]. Immune therapy has continued to evolve and through the use of checkpoint inhibitors, it is now possible to augment the body’s natural ability to fight particular cancers by reversing the effect of immune suppressors produced by the cancer cell to block immune vigilance and elimination of cells undergoing neoplastic transformation, and to stimulate and enhance selective immune-cell-mediated attacks on cancer cells [21,22,23]. While strides are being made on this front, the complexity of cancer and the many forms it takes, presents a large hurdle for identifying markers most appropriate to target with a vaccine or a therapeutic intervention to achieve the maximum effect.

## 4. Targeted Therapies

Further advances in understanding cell biology and the mechanisms underlying a cancer cell’s ability to reproduce, invade, migrate and survive, coupled with the development of proteomic and genetic engineering technology, has further improved our ability to identify and treat individuals at risk for developing cancer [24]. The identification of markers that indicate a predilection for developing cancer has led to the acceptance of prophylactic mastectomy for women who test positively for the genetic markers for BRCA-positive breast cancer, because they have a genetic propensity to undergo oncogenic transformation and are at high risk for developing cancer [25,26]. Knowledge of genetic markers has also improved our ability to treat some cancers in advanced stages of the disease. With viral vectors, the Clustered Regularly Interspaced Short Palindromic Repeats (CRISPR)-Cas9-mediated genome editing technology, poly (ADP-ribose) polymerase inhibitors, and hormonal agents that can modulate cell function or be internalized and have the ability to repair damaged DNA or alter the genetic make-up of cancer cells, it is now possible to deliver treatments more precisely to the source of disease. These can initiate apoptotic cascades, block the ability to reproduce, or guide the production of proteins essential for any number of important functions, including the development of resistance to treatment and the ability to migrate [27,28,29,30]. Unfortunately, cell markers may not be sufficiently different from protein configurations that are expressed by normal cells, which identify them as “self” and part of the host organism [18]. In such instances, as in autoimmune disease, the immune system and enhanced immune therapies do not recognize certain essential parts of the host self and mount an attack on the normal tissues as if they were an invader. The attack on self, which can be as damaging to normal tissue as it is to diseased cells, can be responsible for significant, intolerable and often irreversible adverse effects [31]. In addition, while the activation or inhibition of gene expression has significant potential for treating many forms of cancer, compensatory downstream responses also have the potential to affect resistance and initiate undesired off-target effects [32].

Although the technology that is currently available for producing effective targeted therapies is becoming more readily accessible and adaptable for producing cancer-specific and individualized therapies is improving cancer outcomes and has further reduced the frequency of adverse effects compared with earlier therapeutic methods, the adverse effects observed are not insignificant and remain an impediment to quality of life and limit therapeutic efficacy [33]. Unfortunately, while customizing targeted therapy for individuals can improve the selective delivery and efficacy of treatment, the potential benefits come at a significant cost, affordable to a select few, that is borne by the healthcare system and society as a whole, further limiting their application [9,34].

## 5. An Alternate Approach for Targeting Therapy

That said, despite the recent emphasis and advancements being made in managing cancer with the implementation of targeted therapies and nanotechnology to improve drug delivery [9], cancer remains second only to heart disease as the leading cause of death in the United States [35]. Based on the history of progress in cancer therapy, it is clear that due to the complexity of the disease [9], standalone treatments, while important, have been inadequate to meet the challenge of providing a cure. By contrast, major advances in treatment efficacy have been realized when therapeutic strategies designed to approach the problem in creative ways have been applied in a well-orchestrated fashion to achieve a common goal, and advances associated with the introduction of new and unique approaches to treatment have often been responsible for greater progress than generally modest improvements that are observed with continued refinement of standard approaches [36]. Perhaps we should be open to the idea that there may be other “roads that will lead us to Rome” [37].

**Figure 1:**
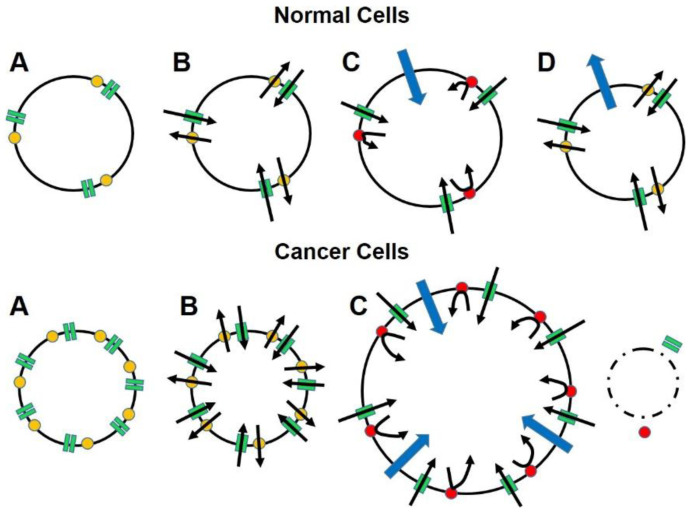
The diagram illustrates how advanced cancers that greatly over-express the conserved and ubiquitous sodium channel/sodium pump mechanism that is essential for cell function and survival, carry their own means of destruction and that by manipulating this mechanism, cancer cells can be eliminated without affecting normal cells. Panels A show that relative to normal cells, cancer cells greatly over express voltage-gated sodium channels (VGSCs; green rectangles) and Na^+^, K^+^-ATPase (yellow dots) more than even highly-expressing normal cells (e.g., nerve and muscle). Panels B show that the functional ratio of VGSCs to sodium pumps is maintained in cancer cells to ensure that the influx of sodium ions (inward oriented black arrows) that occurs down a concentration gradient when the membrane is depolarized and the channels are open can be rapidly reversed (outward oriented black arrows), thereby restoring normal resting membrane potential and intracellular sodium ion concentrations. Panels C show that when the sodium pumps are blocked (red dots) sodium ions enter the cells in direct relation to the number of VGSCs but cannot be returned to the extracellular space. Water enters the cells osmotically (blue arrows) to dilute the intracellular sodium concentrations, causing cell swelling. Panels D show that the amount of water that enters the cancer cells exceeds the cell membrane’s capacity to comply, resulting in cell lysis. In normal cells, the sodium pumps return to normal functioning when the blocking agent clears. The smaller amount of water follows the sodium ions back to the extracellular space, returning the cells to normal configuration and functioning.

In the absence of a preventive vaccine, we offer for consideration a novel and fundamentally different approach, both in principle and design, for treating advanced-stage carcinoma that exploits a basic biological mechanism for survival. The process, called “targeted osmotic lysis (TOL)”, takes advantage of the interdependent, sodium channel/sodium pump alliance that is present in the cells of all animals and is essential for cell communication and survival because of its role in maintaining membrane potential and cellular homeostasis [38,39]. TOL technology is based on the observation that many epithelially-derived cancers over-express voltage-gated sodium channels (VGSCs) and Na^+^, K^+^-ATPase, a feature that confers an enhanced ability to invade normal tissue and to metastasize and is found to be exceedingly prominent in advanced disease, and that the expression of VGSCs in the cancer cells is directly related to the level of malignancy [38,40,41,42]. Although blocking the expression or impeding the function of VGSCs by pharmacologic means has been shown to slow tumor growth and reduces metastasis, these agents leave the original tumor intact and are associated with variable adverse effects imposed on cells that normally express VGSCs [43,44,45,46,47,48,49,50,51,52,53,54,55,56,57,58]. Unlike most targeted technologies that selectively deliver lethal therapies to targeted cells by identifying cellular markers unique to the specific cancer cells, TOL enhances, rather than impedes, VGSC marker functionality, thereby greatly increasing the influx of sodium while simultaneously preventing extrusion of these ions by blocking the sodium pumping mechanism with a cardiac glycoside (Figure 1). Because water passively follows sodium by osmosis and possibly through aquaporins [59], the cells swell beyond their capacity to comply, resulting in cell lysis. Normal cells, even highly-expressing excitable cells (e.g., nerve and muscle) are spared from damage because sodium channel expression in normal tissues is significantly less than that found in most advanced carcinomas. Less sodium, and consequently less water, enters normal cells precluding significant cell swelling and lysis. Unlike destructive therapies that deliver irreversible, lethal agents that destroy all recognized cells, whether malignant or normal, TOL only lyses highly malignant cells that are set apart from normal by the up to 50× greater expression of VGSCs than normal cells [60]. Although TOL affects all cells during active treatment, the Na^+^, K^+^-ATPase blockade is reversible, thus allowing normal cells that do not take on enough water for lysis to return to normal when the cardiac glycoside is released from the receptor and metabolized without producing significant morbidity in the patient.

## 6. Proof-of-Concept Validation

Several studies have been conducted to date to support the initial proof-of-concept for TOL as a potential broad-spectrum treatment for many advanced carcinomas. Initial studies in vitro using immunocytofluorescence were able to confirm the enhanced expression of VGSCs in immortalized breast cancer cell lines [41,42], and that the level of expression correlates directly with the level of malignancy. The TOL effect was demonstrated when treating cells from human breast, lung, prostate, and colon cancer [38,42] and murine triple-negative breast cancer cell lines [38]. It was further shown that time to lysis, using a pulsed electric current (1V DC, 15 pulses per second) delivered with electrodes placed in the vicinity of MDA-MB-231, triple-negative breast cancer cells incubated in ouabain or digoxin, correlates directly with VGSC expression and is dependent on the presence of sodium in the media [38]. Cell lysis was not observed when the electric current was applied to glycoside-treated cell lines derived from normal tissues or malignant cells that were untreated or treated with a drug or stimulation only.

In vivo, high VGSC expression has also been observed using immunohistochemical analysis of tissues taken from ectopic murine and human triple-negative breast cancer xenografts or homografts in Nu/J immune-compromised and BALBc immune-competent mice. TOL has also been shown consistently to be effective in reducing tumor size by 35–45% from baseline (maximum 80–100% reduction in 3 mice), in decreasing the rate of growth, and increasing the survival of mice that serve as hosts to ectopic murine and human triple-negative breast cancer xenografts or homografts treated with digoxin and exposed to pulsed magnetic or electric fields compared to grafts treated with vehicle or a drug or stimulation alone [39]. Despite the effect of TOL on malignant cells, there has been no demonstrable change noted in normal renal, hepatic, dermal, neural and muscle tissues. TOL efficacy has also been observed in dogs and cats when treating a variety of advanced carcinoma, e.g., nasopharyngeal adenocarcinoma, bronchoalveolar carcinoma and metastatic anal gland carcinoma (preliminary observations). In addition to the comparable histopathologic effects on malignancies, 75–90% tumor necrosis extending beyond typical areas of central necrosis, and the lack of damage to normal tissues, it has been possible to note a lack of aversive behavioral signs during and immediately after treatment with TOL and consistent observable, albeit subjective, improvements in appetite, energy and interactive behavior. 

**Figure 2:**
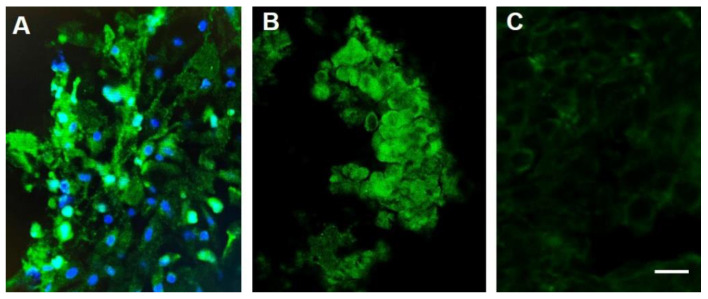
Sodium channel labeling in biopsy samples obtained from a patient with stage IIB squamous cell carcinoma (SCCA) of the cervix (**A**) and a companion canine (**B**,**C**) before (**A**,**B**) and after (**C**) treatment with TOL. The photomicrographs depict the immunohistochemical labeling of VGSCs (green) in a biopsy of the cervical malignancy (**A**) and a canine nasopharyngeal adenocarcinoma (**B**), before (**A**,**B**) and after (**C**) treatment with TOL. Nuclei are counterstained with DRAQ5 (blue). Note that the number of cells in a post-treatment biopsy sample obtained after a single treatment of the canine adenocarcinoma with TOL (**C**) that highly express VGSCs is significantly reduced. The number of cells that had expressed fewer VGSCs and pumps pre-treatment were unaffected and comprised the remaining amount of tumor. Calibration bar in C = 50 µm.

**Figure 3:**
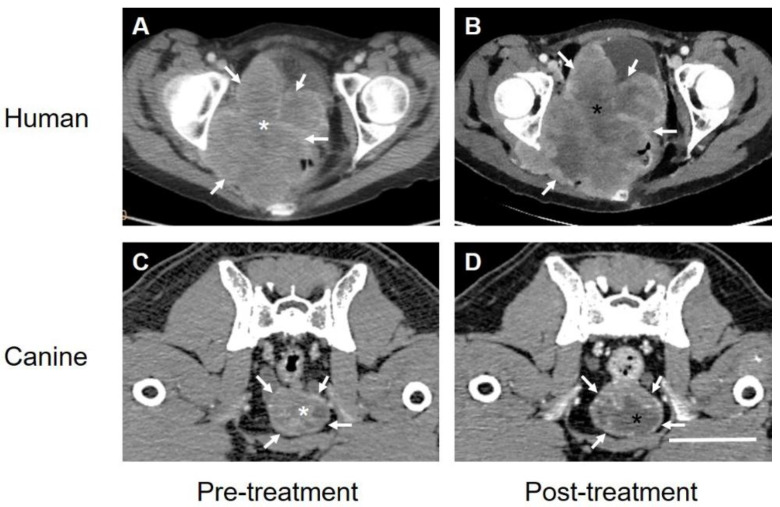
The computer tomographic images of the Stage IIB SCCA of the cervix(A,B) obtained from the patient in Figure 2 and a carcinoma of the prostate obtained from a companion canine (**C**,**D**) before (**A**,**C**) and 3–5 days after (**B**,**D**) treatment with TOL. The images before and after treatment were chosen to depict the respective tumors at levels as closely similar as possible. White arrows indicate points on the surface of the respective mass. Note that the relative size of both types of carcinoma, in the human and the dog post-treatment appears similar if not slightly larger than prior to treatment. By contrast, areas of hypodensity observed within the tumor mass appear larger and much more prominent after treatment (black asterisks) compared to pretreatment (white asterisks) with the region of interest (ROI) measurements of Hounsfield unit densities decreasing from 70 to 56 HU by 3 days post-treatment. Additional region of ROI reference measurements were made for each scan over pelvic musculature revealing values of 127 HU (**A**,**C**) and 120 HU (**B**,**D**), respectively. Calibration bar in D = 5 cm.

Most recently, similar observations related to VGSC expression and response to treatment have been made in a human patient that was allowed a single round of treatment with TOL for late-stage squamous cell carcinoma of the cervix under an Emergency Use protocol [59]. Consistent with the anthropomorphic interpretation of animal responses to treatment, the patient expressed no pain or discomfort related to the administration of treatment and observed increased appetite and a subjective improvement in energy and activity levels and cognitive ability following treatment. The results of the immunohistochemical analysis of VGSC expression (Figure 2) and the imaging (Figure 3) results were similar to those observed in companion animals and consistent with the observations of increased survival in animals, the patient’s 9-week post-treatment survival following a single round of treatment exceeded expectations beyond the days to 2 weeks anticipated when the treatment was requested.

Summary. We argue that TOL is an alternate approach to targeted therapy that may be able to provide a major step forward in improving the level of care for advanced stage carcinoma and warrants further investigation. We propose that TOL is worthy of consideration because (1) it will likely be able to mitigate many limitations associated with current treatment options [60], (2) because TOL seems to be most effective when VGSCs are most highly expressed [38], and VGSCs expression in the cancer cells is greatest in the most malignant and advanced forms of a carcinoma, TOL is likely to be most effective for treating advanced stage cancers that are responsible for most of the over 600,000 cancer deaths seen each year in the U.S. alone [35], (3) because of the conserved nature, the ubiquitous distribution and the consistent functional characteristics of the sodium channel/sodium pump mechanism throughout the animal kingdom, TOL technology has the potential to provide broad-reaching treatment for many forms of advanced carcinoma, and (4) because of the broad coverage, the cost of research and development of the technology and delivery of treatment can be shared by a significant portion of the population, making it likely that the cost of treatment with TOL will be more affordable than currently available therapies. The potential adverse effects associated with tumor lysis syndrome, a complication associated with the elimination of large tumor masses that should be anticipated, to date, have not been observed following administration of TOL. This adverse effect is, however, subject to prophylactic measures of fluid hydration and treatment with allopurinol or hemodialysis. Avoidance of potential concerns related to the use of TOL to treat cancer in patients with co-morbid chronic inflammatory conditions that due to chronic inflammation may have tissues that over-express VGSC [61,62,63,64,65,66] requires further study.

## 7. Conclusions

We are fortunate that current methods for treating malignancy have, in some cases, been able to provide a cure, and in others, have increased survival. Yet even with the current targeted therapies, many patients still die in the prime of their lives, suffer from serious comorbidities and experience intolerable adverse effects that limit the possibility of a cure, and often endure a significant compromise in quality of life that may extend far beyond the period of treatment. The evidence to date, drawn from studies conducted on several forms of cancer performed in vitro and in vivo in several mammalian species supports the proposal that TOL, while needing additional refinement to improve efficacy and minimize resistance to treatment, has the potential to provide a safe, well-tolerated and effective treatment for advanced carcinomas that offers a possibility to extend the quantity of life without compromising quality. Whether TOL can be used as a standalone therapy or as part of a multimodal treatment algorithm before or after surgical resection and/or radiation, before or after chemotherapy, immune therapy or genetic engineering procedures, is yet to be determined. 

## 8. Patents

A patent for the technology described in this manuscript entitled, Targeted Osmotic Lysis of Cancer Cells—File No. 11M01 (Serial No. 13/552,909) Paul, D.J. and Gould, H.J., III was allowed on 30 December 2014.
